# Diagnostic utility of ESR1 mutation detection in liquid biopsy of metastatic breast cancer patients

**DOI:** 10.1007/s00428-024-03942-1

**Published:** 2024-10-11

**Authors:** Maria Sandbothe, Britta Hasemeier, Elisa Schipper, Nora Schaumann, Hans Kreipe, Ulrich Lehmann, Stephan Bartels

**Affiliations:** Institute of Pathology, Hannover Medical School, Carl-Neuberg-Str. 1, 30625 Hannover, Germany

**Keywords:** Breast cancer, Metastasis, Elacestrant, *ESR1*, Liquid biopsy

## Abstract

Molecular analysis of circulating cell-free DNA (cfDNA) extracted from peripheral blood plasma samples of metastatic breast cancer (BC) patients is of rising interest to find optimal therapeutic strategies. Detection of emerging resistance mutations against endocrine therapy is possible with this approach. Here we present the applicability of a laboratory-developed NGS assay in molecular pathology routine diagnostic, covering four genes with therapeutic (*ESR1*, *PIK3CA*, *ERBB2*) and prognostic (*TP53*) consequences in metastatic BC. We analyzed 162 liquid biopsy samples and 25 corresponding metastases from metastatic BC patients. In the liquid biopsies, we detected *ESR1* mutations in 42 cases (25.9%) and *ERBB2* mutations in six cases (3.7%), arguing for a change in therapy to fulvestrant, elacestrant, or neratinib. Furthermore, 17 cases had detectable *TP53* mutations, associated with resistance against endocrine therapy. We conclude that liquid biopsy testing is a noninvasive, sensitive, and helpful method to optimize therapeutic decisions in metastatic BC.

**Supplementary Information:**

The online version contains supplementary material available at 10.1007/s00428-024-03942-1.

## Introduction

About 20–30% of hormone receptor positive breast cancers (BCs) show local or distant recurrence after endocrine treatment [1]. Many of these metastatic BC cases, treated with tamoxifen or aromatase inhibitors (AI), acquire a secondary resistance against hormone blockade [2]. Ligand-independent, constitutive estrogen receptor α (ER) activity, caused by the acquisition of *ESR1* gene mutations in the ligand-binding domain, represents a resistance mechanism of tumor cells against anti-estrogenic therapy [3]. In case of early *ESR1* mutation detection, patients can benefit from switching the treatment to fulvestrant. Progression-free survival in a recent phase 3 trial was 11.9 months in the fulvestrant and palbociclib group versus 5.7 months in the AI and palbociclib group [4]. Furthermore, with elacestrant, a new oral ER degrader is available. In heavily pre-treated patients, 12-month PFS of *ESR1* mutated cases was 22.3% with elacestrant versus 9.4% in the standard-of-care group [5]. A noninvasive alternative to tissue biopsies of metastatic sites is the mutation detection in circulating free DNA (cfDNA) from tumor cells extracted from blood plasma samples (liquid biopsy). Here we present real-world data from *ESR1* mutation testing in external liquid biopsies in molecular pathology routine.

## Material and methods

### Patient samples

Unselected peripheral blood samples from metastatic breast cancer patients were collected in hospitals and oncology practice units in northern Germany from January 2023 to June 2024; 157 from 162 (96.9%) samples were received after the European Medicines Agency (EMA) approval of elacestrant in September 2023. For 143 (88.3%) patients, the clinical information indicated that they were suffering from progressive disease under endocrine therapy. For 19 (11.7%) patients, no clinical data was available. Cell-Free DNA BCT® tubes (Streck, La Vista, USA) with 10 ml blood were sent at room temperature to the Institute of Pathology at the Hannover Medical School. Recently taken formalin-fixed and paraffin-embedded (FFPE) tissue specimens from metastatic sites were available for 25 patients.

### Nucleic acid extraction

Plasma was separated from blood cells by centrifuging three times at 1000* g* for 20 min immediately upon arrival. Separated blood plasma was stored at − 80 °C. Up to 5 ml plasma was used for circulating free DNA (cfDNA) extraction with the QIAamp circulating nucleic acid kit (Qiagen, Hilden, Germany) following the recommendations of the manufacturer. The cfDNA was eluted in 40 µl Elution Buffer in a DNA LoBind tube and stored at − 20 °C. DNA extraction from FFPE tissue specimens of metastatic sites was performed as described [6].

### Sequencing and data interpretation

A custom designed laboratory-developed NGS panel containing 157 amplicons, covering the complete coding sequence of *ESR1* (NM_001122740.2), *PIK3CA* (NM_006218.4), *ERBB2* (NM_004448.4), and *TP53* (NM_000546.6), was used for library preparation with the Ion AmpliSeq™ Library kit plus (Thermo Fisher Scientific, Waltham, USA). The panel performance was validated with the ESR1 Reference Set 1% AF cfDNA (SensID, Rostock, Germany, supplementary Table 1). Sequencing was performed on an Ion S5 prime instrument with 2,500,000 or 500,000 reads per sample (approximately 15,000 reads or 3000 mean depth) for liquid biopsies or FFPE material, respectively. Evaluation of sequencing data and variant annotation was performed with the ANNOVAR software and database tools [7].

## Results

**Fig. 1:**
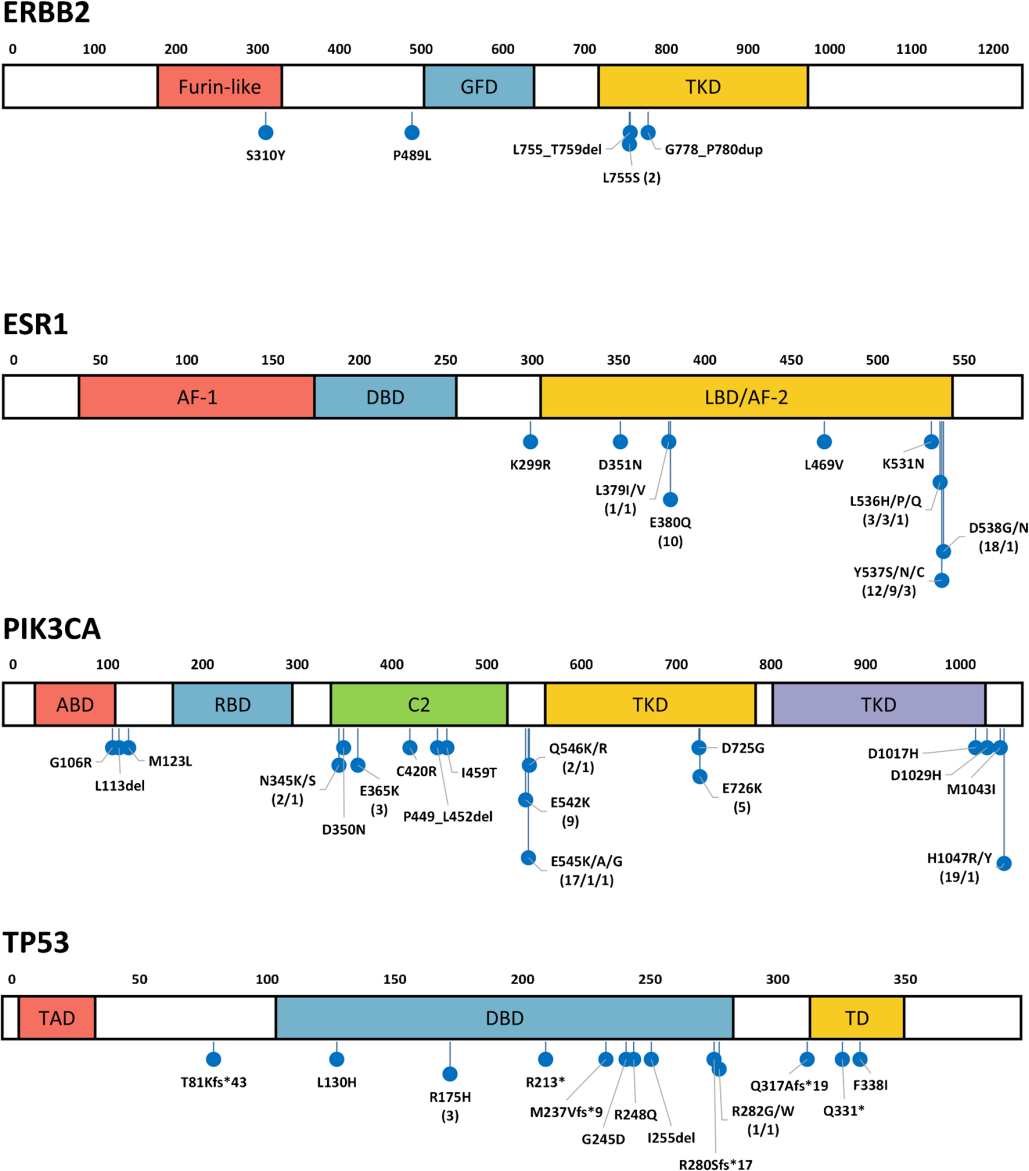
Distribution of detected mutations in 162 liquid biopsy samples within functional domains of the genes *ERBB2*, *ESR1*, *PIK3CA*, and *TP53*. If a specific mutation was found in more than one sample, the total number is given in parentheses

**Fig. 2:**
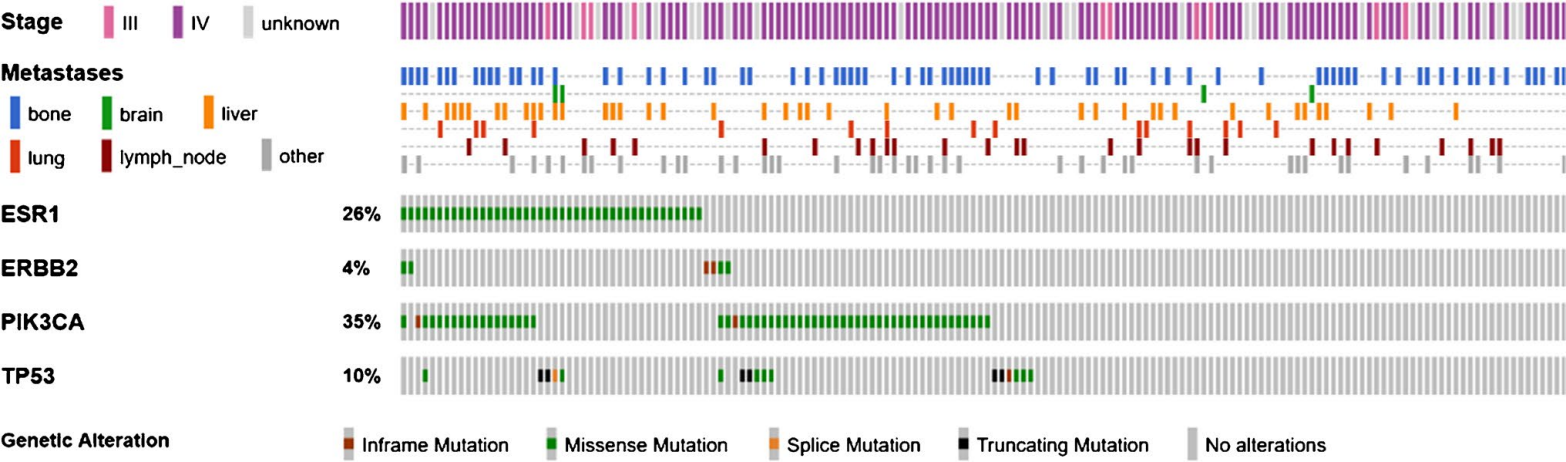
Clinical features and gene mutations detected in liquid biopsy samples of 162 BC patients (created in https://www.cbioportal.org/oncoprinter). For details, see supplementary Table 2

**Fig. 3:**
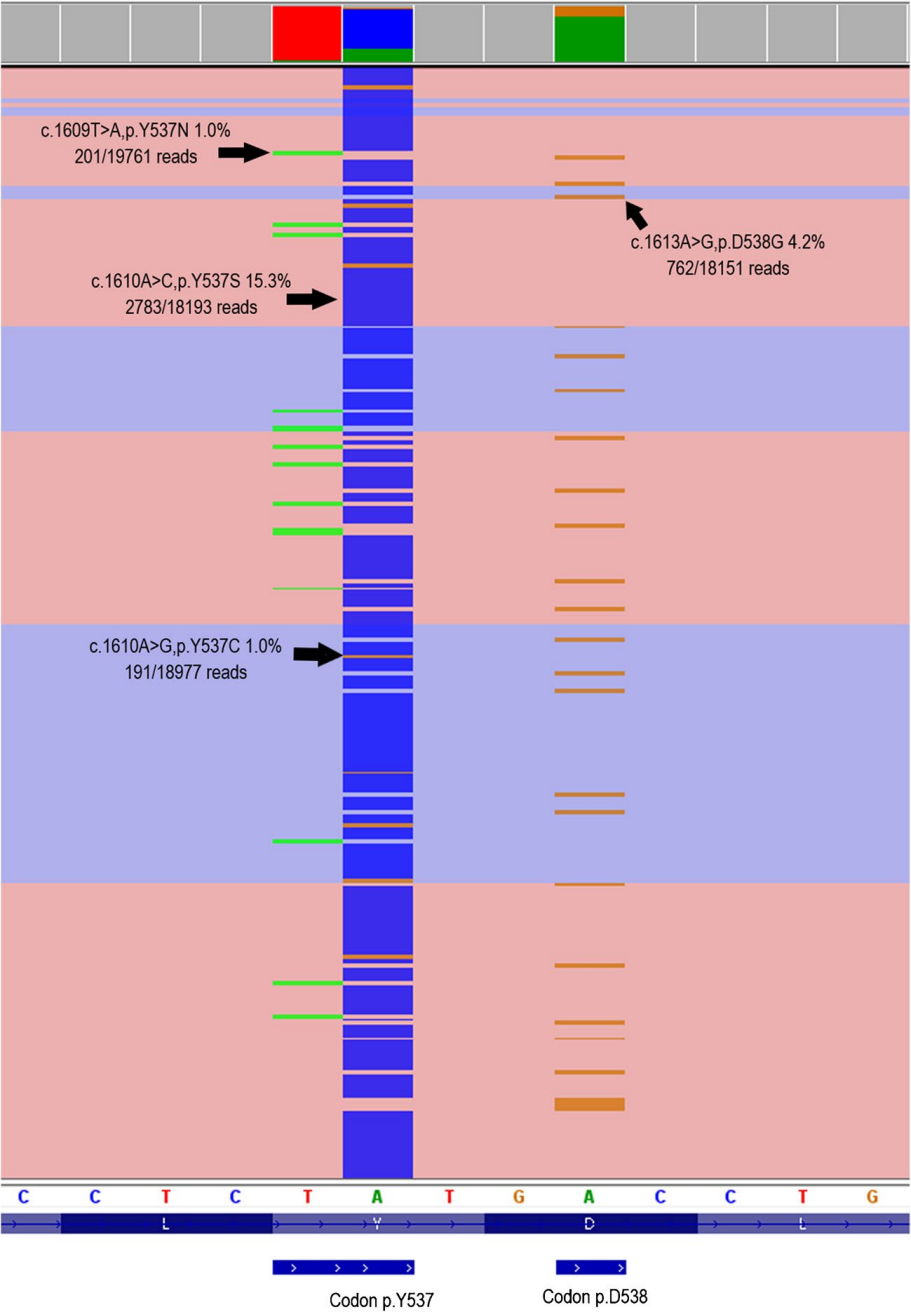
Four sub-clonal *ESR1* hotspot mutations *in trans* (p.Y537N/S/C and p.D538G) from case no. 28. The p.E380Q mutation in exon 6 is not shown

We analyzed liquid biopsy samples from in total 162 patients with metastatic BC to identify mutations in *ESR1*, *PIK3CA*, *ERBB2*, and *TP53*. The mean cfDNA amount extracted from 5 ml blood plasma was 124.6 ng; the median was 74.0 ng (details for all samples in supplementary Table 2). There was a positive correlation of mutation calls with a higher amount of cfDNA (supplementary Figure 1). In 88 patients (54.3%), we detected a total of 162 gene mutations (Fig. 1, supplementary Table 2). In 30 patients (18.5%), we detected co-mutations in two or three of the investigated genes (Fig. 2). *ESR1* mutations could be detected in 42 cases (25.9%). The lowest and the highest detected allele frequency (AF) of an *ESR1* hotspot mutation was 0.2% and 70.9%, respectively (both p.E380Q, case no. 61 and 75 in supplementary Table 2). Thirteen cases showed multiple *ESR1* mutations, indicating sub-clonal diversity (Fig. 3). The recently described *ESR1* p.F404L/I/V fulvestrant resistance mutations [8] are not present in our cohort. *PIK3CA* mutations occurred most frequently (56 cases, 34.6%), among them 16 cases with a combined *PIK3CA/ESR1* mutation. In six cases (3.7%), *ERBB2* mutations in exon 8, 12, 19, or 20 were detectable. *TP53* mutations were present in 17 cases (10.5%), mostly co-mutated with *ERBB2*, *ESR1*, or *PIK3CA*. The mean turnaround time in the molecular laboratory was 5.9 working days (range 3 to 11 days), which is acceptable for NGS panel sequencing including data analysis.

Information about the location of metastatic sites was available for 130 of 162 patients (80.2%, supplementary Table 2), but only for 25 patients FFPE material from metastases was available for sequencing (supplementary Table 3). For 15 patients, we detected the same mutations in liquid biopsies and metastases. For five patients, we did not detect any mutations in the liquid biopsy while there were eight detectable mutations in the metastatic site DNA, among them two *ESR1* hotspot mutations. Conversely, also for five patients, we detected seven mutations in the liquid biopsy that were not detectable in the metastatic site DNA. Of note, all of these were *ESR1* hotspot mutations. Two of these five patients had several sub-clonal *ESR1* mutations in the liquid biopsy from which one was also detectable in the metastatic site DNA. Three of these five patients had a positive *ESR1* mutation call exclusively in the liquid biopsy.

## Discussion

Since market-entering of the oral ER degrader elacestrant at the end of 2023, liquid biopsy testing of metastatic BC increased immensely. In the laboratory-developed NGS panel employed in this study, the four disease relevant genes *ESR1*, *PIK3CA*, *ERRB2*, and *TP53* were tested. Detection of an *ESR1* mutation in metastatic BC should be followed by changing anti-estrogenic therapy to fulvestrant or elacestrant [4, 5]. *PIK3CA* mutations are frequent in BC and therefore a useful biomarker to ensure that cfDNA originating from tumor cells is present. Furthermore, *PIK3CA* mutations are possible targets for alpelisib [9] or capivasertib [10]. *ERBB2* mutations are present in bone marrow metastases from lobular BC cases, and are possibly targetable with anti-HER2 therapy [6]. *TP53* mutations are associated with primary endocrine resistance and might not be adequately treated with endocrine therapy alone [11].

In a recent study on tissue biopsies from breast cancer metastases, we detected *ESR1* hotspot mutations in 78 of 521 (15.0%) samples [12]. In the current study, using liquid biopsy, we detected *ESR1* mutations in 25.9% of cases. In a recent meta-analysis of 16 studies with in total *n* = 2744 patients, the overall incidence of *ESR1* mutations was 23% (ranging from 11 to 55%) [13]. Differences in the mutation frequency are possibly not caused by the testing material (tissue or liquid), but by the selection of samples in the studies. For example, in *n* = 194 ER-positive bone marrow BC metastasis, we found 14% *ESR1* mutation frequency [14], whereas lung and liver metastasis showed 26.3% and 31.3% *ESR1* mutation frequency, respectively [12]. Nearly 97% of the patient samples analyzed in the current study were collected after the EMA approval of elacestrant. This strong increase reflects the urgent clinical need of new therapeutic approaches in anti-endocrine therapy in metastatic BC.

Our comparison of cfDNA and metastatic site DNA from 25 patients shows that *ESR1* hotspot mutations may be missed in both sample materials. In three patients, an *ESR1* mutation was detected only in the liquid biopsy cfDNA, whereas two patients had the positive mutation call only in metastatic site DNA. False-negative results in liquid biopsy analysis can be caused by contamination with white blood cell DNA or absence of tumor DNA at time of blood collection. Further, the sample transfer from external clinics and oncology practice units to the molecular pathology laboratory is prone to errors, e.g., transfer time too long. Clear instructions and regular consultation are necessary to reduce handling errors which can lead to false-negative results [15]. Nevertheless, especially in patients with multiple metastatic sites, liquid biopsies are the preferred material for molecular analysis, because the genetic heterogeneity of metastatic sub-clones is potentially represented in the cfDNA. However, in case of negative results from a liquid biopsy, the analysis of a most recent metastasis specimen should be considered if possible.

In conclusion, our results demonstrate that *ESR1* detection in liquid biopsy is feasible under routine conditions identifying important therapy-changing predictive biomarkers in a substantial portion of metastatic BC patients. To the best of our knowledge, this is the first study which demonstrates the feasibility and diagnostic gain of *ESR1* mutation detection in real-world liquid biopsy outpatient samples since the approval of elacestrant.

## Supplementary Information

Below is the link to the electronic supplementary material.

Supplementary file1 (XLSX 13 KB)

Supplementary file2 (XLSX 271 KB)

Supplementary file3 (XLSX 119 KB)

Supplementary file4 (TIF 410 KB)

## Data Availability

Data generated or analyzed during this study are included in this published article.
