# Supplementary figures and images for: Diagnostic utility of ESR1 mutation detection in liquid biopsy of metastatic breast cancer patients
Source: Virchows Arch. 2024 Oct 11;487(4):895–9. doi: 10.1007/s00428-024-03942-1 (PMC12546375; doi:10.1007/s00428-024-03942-1)

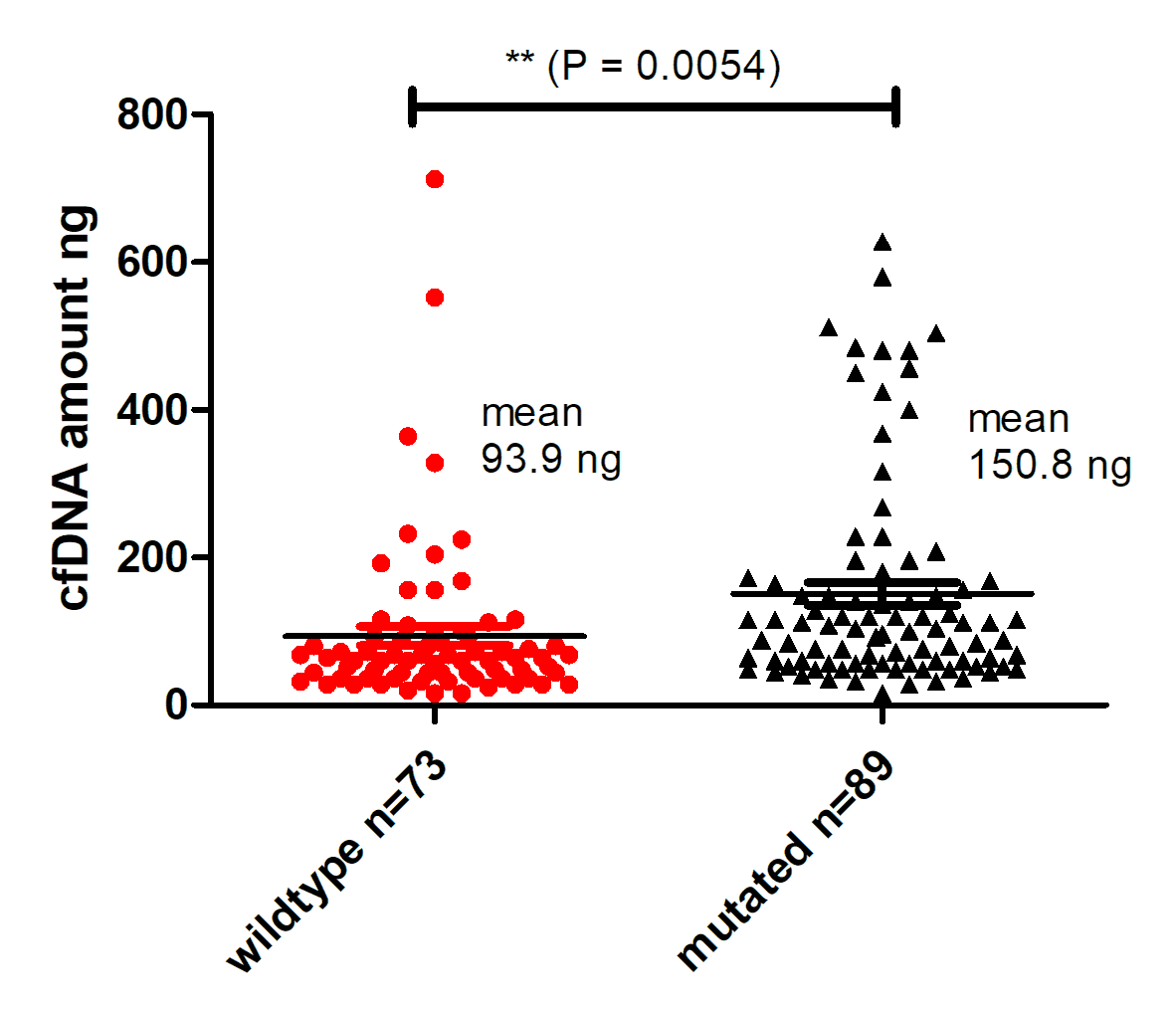

Supplement: Supplementary file 4 — Supplementary file4 (TIF 410 KB) [file 428_2024_3942_MOESM4_ESM.tif]
